# The role of non-protein-coding RNAs in ischemic acute kidney injury

**DOI:** 10.3389/fimmu.2024.1230742

**Published:** 2024-02-08

**Authors:** Fatemeh Sabet Sarvestani, Afsoon Afshari, Negar Azarpira

**Affiliations:** ^1^ Shiraz Transplant Research Center, Shiraz University of Medical Sciences, Shiraz, Iran; ^2^ Shiraz Nephro-Urology Research Center, Shiraz University of Medical Sciences, Shiraz, Iran

**Keywords:** noncoding RNAs, miRNAs, lncRNAs, circular RNAs, ischemia, acute kidney injury

## Abstract

Acute kidney injury (AKI) is a condition characterized by a rapid decline in kidney function within a span of 48 hours. It is influenced by various factors including inflammation, oxidative stress, excessive calcium levels within cells, activation of the renin-angiotensin system, and dysfunction in microcirculation. Ischemia-reperfusion injury (IRI) is recognized as a major cause of AKI; however, the precise mechanisms behind this process are not yet fully understood and effective treatments are still needed. To enhance the accuracy of diagnosing AKI during its early stages, the utilization of innovative markers is crucial. Numerous studies suggest that certain noncoding RNAs (ncRNAs), such as long noncoding RNAs (lncRNAs), microRNAs (miRNAs), and circular RNAs (circRNAs), play a central role in regulating gene expression and protein synthesis. These ncRNAs are closely associated with the development and recovery of AKI and have been detected in both kidney tissue and bodily fluids. Furthermore, specific ncRNAs may serve as diagnostic markers and potential targets for therapeutic interventions in AKI. This review aims to summarize the functional roles and changes observed in noncoding RNAs during ischemic AKI, as well as explore their therapeutic potential.

## 1 Introduction

**Figure 1:**
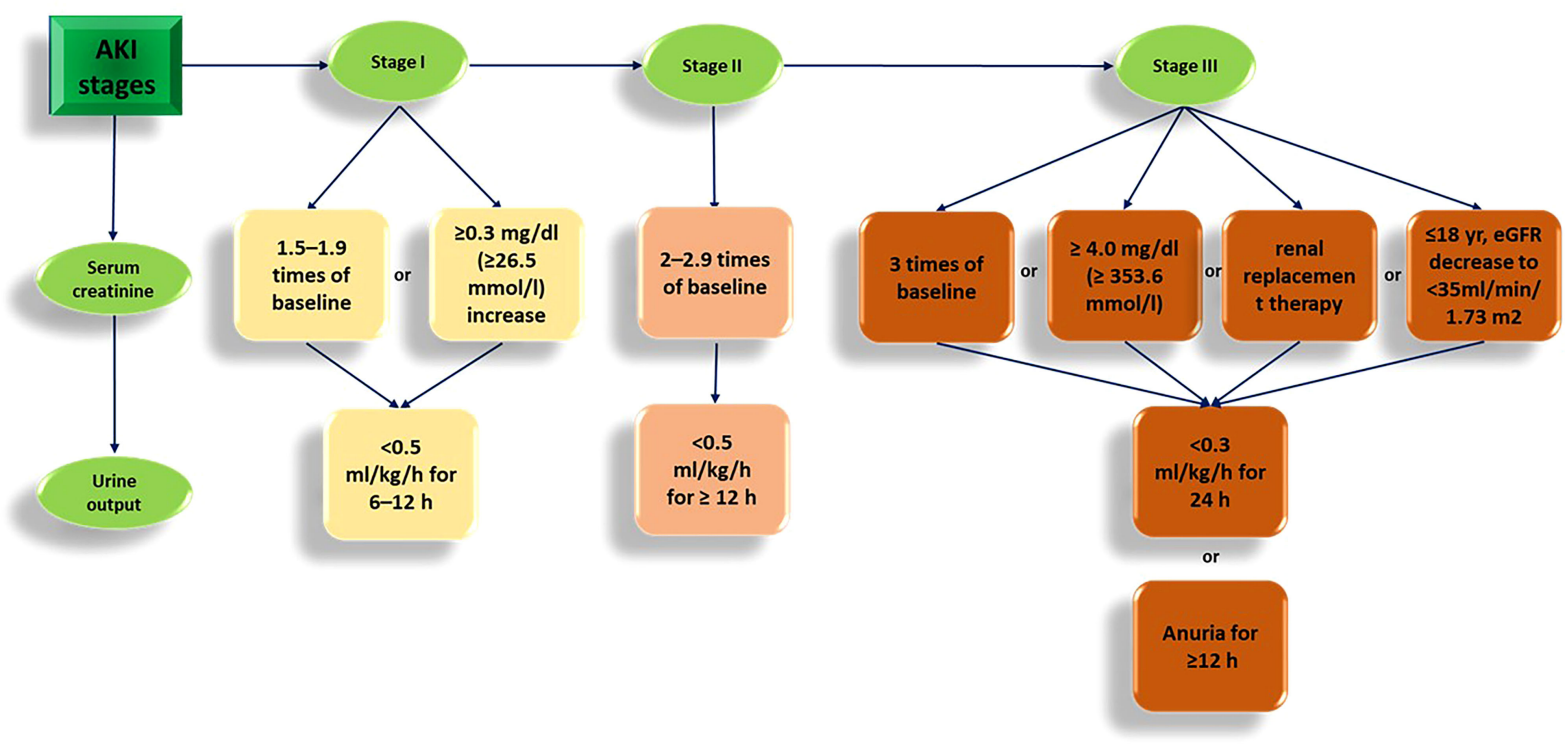
The KDIGO guidelines for AKI in 2012.

In the past, acute kidney injury (AKI) was referred to as “acute renal failure.” It is identified by a sudden decline in kidney function, usually indicated by a significant rise in serum creatinine (Cr) levels within a brief timeframe ranging from hours to weeks, or less than 3 months (1). This condition is prevalent in hospitalized patients, up to 10-20% of all patients and up to 50% of those in intensive care units (ICU). It is related to significant morbidity, mortality, and healthcare costs (2). The main causes of AKI comprise ischemia/reperfusion injury (IRI), sepsis, and exposure to nephrotoxins such as cisplatin and contrast media (3). The 2012 Kidney Disease Improving Global Outcomes (KDIGO) guidelines define AKI considering levels of detecting Cr in the serum and urine of patients. When AKI conditions remain more than seven days (**Figure 1**), it is classified as AKI (4). AKI complicates patient recovery and is associated with volume overload, electrolyte disorders, drug toxicity, and uremia and increases the risk of cardiovascular events and the progress of chronic kidney disease (CKD) events (5, 6).

As feedback of hypoxia and reperfusion, IRI typically triggers a strong inflammation and oxidative stress, that disrupts the organ function. In the case of renal IR, this can result in AKI and can contribute to increased morbidity and mortality in various types of injuries (7). Apart from supportive measures, there are currently no proven therapies available for efficient treatment or diagnosis of ischemic AKI. However, experimental researches have offered a better perception of the pathophysiology of ischemic AKI, and there is hope of finding effective therapies or even inhibitory strategies which may emerge in the next years (8).

Noncoding RNAs (ncRNAs), which are transcribed from the genome, do not function through the production of proteins, but instead operate at the RNA level. Several types of ncRNAs exist, some of the most studied types are; microRNAs (miRNAs), long noncoding RNAs (lncRNAs), circular RNAs (circRNAs), small nucleolar RNAs (snoRNAs), small nuclear RNAs (snRNAs), small interfering RNAs (siRNAs) transfer RNAs (tRNAs), and ribosomal RNAs (rRNAs) (9–11).

For instance, LncRNA-NEAT1 increased expression and decreased in its miRNA target, miR-27-3p, in ischemia‐induced AKI patients have been introduced (12). A different research study emphasized the significance of ciRs-126, which had the greatest degree of change in comparison to both healthy and diseased controls. In this regard, ciRs-126 was observed to act as a sponge for miR-126-5p using bioinformatics, and the latter was found to be significantly suppressed in patients with AKI as well as in endothelial cells exposed to hypoxia (13).

Patients with AKI still experience high morbidity and mortality rates, highlighting the urgent need for urgent and accurate diagnostic and therapeutic strategies. In order to enhance the AKI diagnosis accuracy and identify new therapeutic molecules, researchers have turned their attention to ncRNAs, which have potential in both AKI diagnosis and treatment (14). The trace of these molecules has been detected in the studies related to IRI and AKI. Here, it is tried to gather related data in the current field which believes that might be a hallmark for finding further diagnostic and treatment solution for ischemic AKI.

## 2 IRI and AKI physiology in kidney: definition, epidemiology, pathogenesis, and morphogenesis

IRI occurs when there is a sudden and temporary impairment in the blood flow to a specific organ. IRI refers to the constriction, accompanied by the restoration and re-oxygenation of blood flow to an organ. This process can cause tissue damage and trigger an inflammatory response, including the activation of leukocytes, reactive oxygen species (ROS), cytokines, and chemokines (15, 16). IRI can happen subsequent to infarction, sepsis, and organ transplantation, and it can worsen the tissue damage. In the kidney, IRI can lead to AKI (17, 18). It should be considered that reperfusion itself can cause significant additional cellular injury, despite being crucial to the restoration of kidney function (19).

Following the incidence of IRI, the proximal tubular cells undergo a rapid loss of integrity in their cell membrane and cytoskeleton. This phenomenon leads to the displacement of adhesion molecules and Na+/K+-ATPase, thereby causing a loss of polarity in the normally highly polar epithelial cells. The cytoskeletal changes contribute to the regulation of cell polarity, cell–cell interactions, and cell-matrix interactions (20).

Incidence of cell death facilitates due to necrosis and/or apoptosis during an increase in the time or severity of ischemia. This phenomenon is continued by recruiting of inflammatory cells and release of proinflammatory molecules such as tumor necrosis factor-alpha (TNF-α), interleukin-1 (IL-1), interleukin-6 (IL-6), and monocyte chemoattractant protein-1 (MCP-1) (3, 16). Activation of these molecules exacerbate the injury of tubular epithelial and endothelial cells of kidney and even intensify the immune responses, increase inflammatory infiltration, and simplify the “inflammatory cascade effect.” Eventually, surviving epithelial cells start migration into the basement membrane denuded areas, divide, replace lost cells, then differentiate to re-establish normal epithelial polarity. Consequently, the pathophysiology of the produced damage involves a multifaceted interaction between the vasculature, the tubules, and inflammation. It is noteworthy that both innate and adaptive immunity has critical role in the primary damage stage, regulation of the inflammatory response, and repair phase (16).

Acute renal failure (ARF) is a common clinical problem characterized by a sudden decline in kidney function, leading to the accumulation of nitrogenous waste molecules such as blood urea nitrogen (BUN) and creatinine (Cr). This condition poses significant challenges due to its increasing prevalence, high treatment costs, limited treatment options, and substantial economic impact on society (21). ARF may be graded as prerenal (efficient response of normal kidneys to hypoperfusion), intrinsic renal (structural injury to the renal parenchyma), and postrenal (urinary tract obstruction) (22).

The most frequent and severe subtype of ARF in hospitalized patients is intrinsic ARF, which can be pathologically linked to acute tubular necrosis (ATN). Thus, intrinsic ARF and ATN are commonly used interchangeably in clinical practice. Despite significant advances in both basic research and medical care, the prediction for intrinsic ARF in the patients still remains bleak, with mortality rates ranging from 40-80% during the intensive care setting. The field has been plagued by two major issues that have hindered progress. The first is the use of more than 20 different definitions for ARF in available studies, extending from subtle increases in serum Cr to requirements for dialysis (23). In an effort to establish a uniform definition encompassing the various aspects of the disease, a new term, AKI, has been suggested (24). AKI is a multifaceted disorder that encompasses various underlying factors and presents with diverse clinical symptoms that can range from a persistent yet slight increase in serum Cr to complete renal failure with a lack of urine production. Therefore, AKI represents a complex clinical entity that demands a comprehensive understanding of its underlying pathophysiology and a tailored approach to diagnosis and treatment (22).

In the clinical setting, the conventional method of assessing renal function involves measuring Cr and urea levels and monitoring urine output. However, research has demonstrated that these markers are inadequate and tardy in diagnosing AKI (25). In clinical practice, renal function is usually assessed by measuring Cr and urea levels and monitoring diuresis. However, diuresis can be influenced by various factors independent of renal function, such as volume depletion or diuretic use. To overcome these limitations, researchers have focused on developing new and more sensitive biomarkers (14). Despite advances in preventive strategies and supportive measures, AKI remains associated with high morbidity and mortality, especially in patients admitted to the ICU. Besides mortality rates, surviving AKI patients are at risk of developing or worsening CKD, which can hasten the development into end-stage renal disease (ESRD) (26, 27).

Moreover, the unadjusted mortality rate for adults experiencing a period of AKI is predicted to be 23.9%, while it is 13.8% for children (2). Moreover, studies have revealed that the risk of in-hospital death increases in a near-linear fashion with the AKI severity (28). An epidemiological investigation conducted in the United States indicates that each year, roughly 600,000 patients are diagnosed with AKI, accounting for 1.9% of total inpatients, with 128,000 of these patients succumbing to the disease annually. Furthermore, a worldwide survey has concluded that AKI is responsible for approximately two million deaths annually (29, 30).

AKI has a multifactorial pathogenesis involving vascular dysfunction, inflammation, and tubular injury. Several cell types and molecular regulators/mediators are responsible for initiating and progressing the disease (3). In response to AKI, the remaining renal tubular cells dedifferentiate and proliferate for replacing injured tubular epithelial cells, restoring normal renal function and tubular integrity. In situations of severe and multiple AKI, kidney repair may be incomplete or maladaptive, leading to renal fibrosis and ultimately, chronic kidney disease (CKD) (AKI-CKD) (6, 31).

Ischemic AKI in humans is characterized by several morphological changes such as, the loss of the proximal tubule brush border, occasional damage to tubule cells, focal areas of proximal tubular dilation, and distal tubular casts. Cellular regeneration can also be observed (22). However, necrosis is not prominent and is confined to the outer medullary regions. Unless a principal glomerular ailment is the source of the ARF, glomeruli usually appear normal. The subtle histologic changes observed in AKI have long been a source of concern given the severe impairment of renal function. However, studies have shown that distal as well as proximal tubules in both ischemic and nephrotoxic AKI display apoptotic cell death (32, 33). The peritubular capillaries also show signs of vascular congestion, endothelial damage, and leukocyte accumulation, which have garnered significant attention (34, 35).

## 3 Ischemic AKI

The pathophysiology of kidney IRI is a complicated process that involves several pathways, including the immune system and metabolic pathway that involves an imbalance between pro- and anti-inflammatory cytokines, leading to the activation of innate immune cells, the release of ROS, and other inflammatory mediators and subsequent renal injury and fibrosis (7, 36).

### 3.1 Ischemic AKI molecular mechanisms

**Figure 2:**
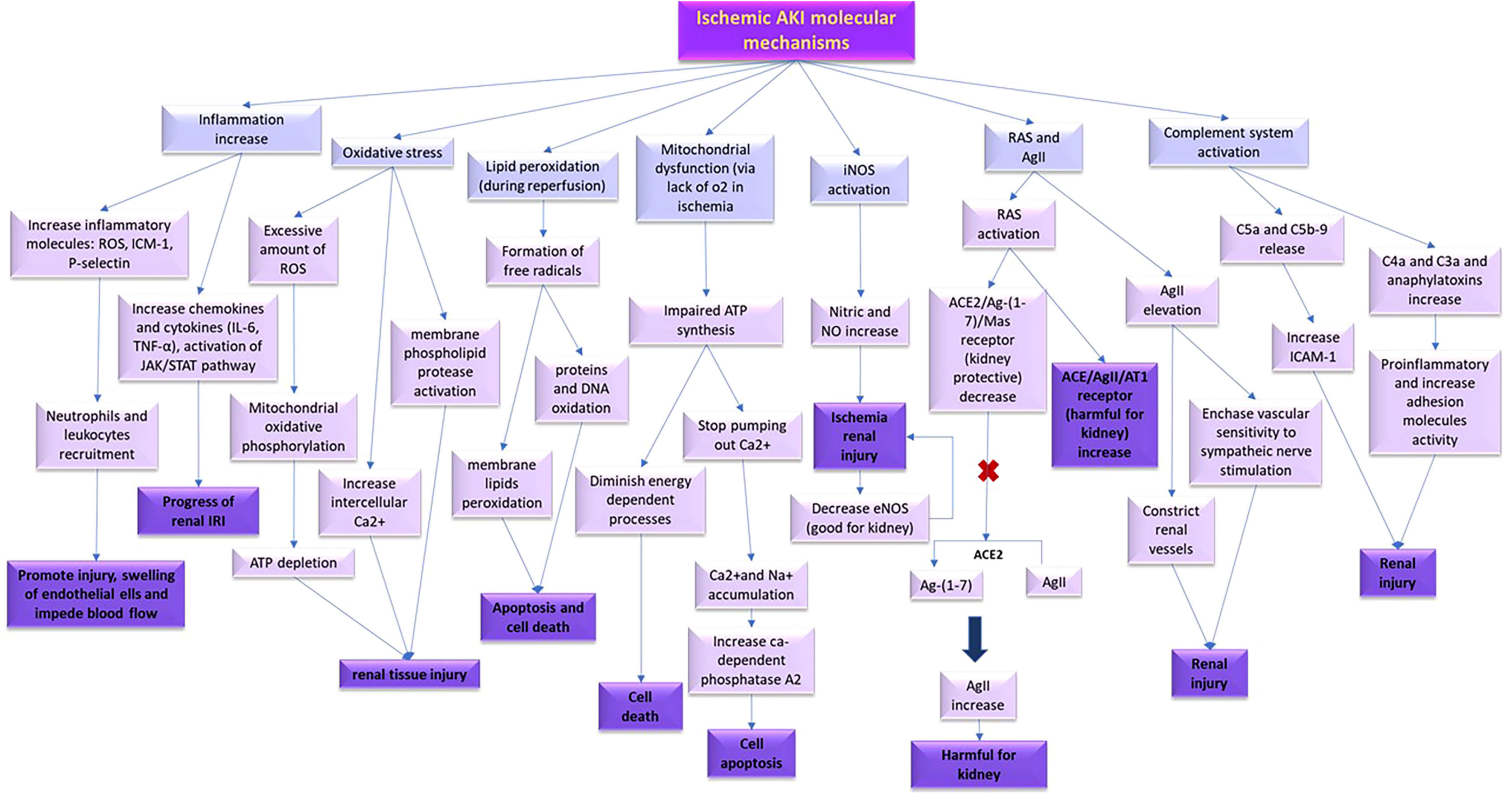
Molecular processes that happen during ischemic AKI.

The current study aims to present an overview of the molecular processes that occur during ischemic AKI, as depicted in **Figure 2**.

#### 3.1.1 Inflammation, leukocytes, and adhesion molecules

During IRI in the kidney, inflammation acts as a prevalent abnormality and plays a crucial role in its pathophysiology by connecting various cell types. It starts an inflammatory cascade that exacerbates renal damage, inhibits inflammatory responses that are potential therapeutic strategies for protecting renal tissue (37, 38). The chemokine molecules regulate the expression of pro-inflammatory cytokines, adhesion molecules, and finally result in the leukocyte infiltration and activation (39). The pathogenesis of renal dysfunction in IRI is influenced significantly by pro-inflammatory cytokines, including IL-6 and TNF-α (40–42). The JAK/STAT pathway is responsible for mediating several pro-inflammatory molecules that contribute to the progression of renal IRI (43).

It is documented that cell adhesion molecules including intracellular adhesion molecule-1 (ICAM-1) and P-selectin, ROS, and inflammatory mediators play a crucial role in recruiting leukocytes and neutrophils toward the tissue harboring ischemia. This results in an increased interaction between leukocytes and endothelial cells, cause damage, and blood flow obstruction (44, 45). Therefore, the activation of inflammatory leukotriene pathway observed in both acute and chronic renal failure during IRI (46, 47). Activation of leukocytes, particularly neutrophils, are important factors in the renal IRI progress (48). Neutrophils exacerbate IRI through releasing different mediators including ROS, cytokines, and proteases (49). Collectively, it appears that inflammation, leukocytes, and adhesion molecules play a significant role during IRI, and any interventions that decrease the inflammatory procedure or prevent infiltrating leukocytes and neutrophils may be appropriate for attenuating the side effects of kidney IRI (7).

#### 3.1.2 Oxidative stress

During IRI, the injured organ generates a considerable amount of ROS, which leads to oxidative stress. This oxidative stress ends in modifications in oxidative phosphorylation in the mitochondria, reduction of ATP, an intracellular calcium increase, and the activation of membrane phospholipid proteases (50).

#### 3.1.3 Lipid peroxidation

The reperfusion phase of IRI can lead to the generation of oxygen free radicals through the restoration of blood flow, which can cause lipid peroxidation (51). The formation of free radicals is the main basis of renal tissue injury due to peroxidation of the membrane lipids and oxidative damages that target proteins and DNA (52). Additionally, IRI pathophysiology might be a result of down-regulation in some of the antioxidant enzymes including catalase, superoxide dismutase, and glutathione peroxidase (53).

#### 3.1.4 Mitochondrial dysfunction

During ischemia, the lack of oxygen suppresses mitochondrial oxidative phosphorylation. The primary target and the main source of intracellular ROS is mitochondria. ATP reduction causes a halt in the pumping of calcium out of the cell, leading to accumulation of it within the cell. As this process is executed through Na/Ca2+ antiporter channel, the Na/K/ATPase pump is not capable of recovering accumulated intracellular sodium (54). Furthermore, an excess of intracellular calcium results from the redistribution of calcium stored in the endoplasmic reticulum and activate some of calcium-dependent enzymes such as phospholipase A2, endonuclease, and proteases, which initiate the process of cellular apoptosis (55). During the reperfusion phase, post-ischemic cell mitochondria are exposed to high levels of calcium and free radicals of oxygen, that gradually worsen mitochondrial function (56).

#### 3.1.5 Nitrite and nitric oxide

NO, is produced by endothelial cells and at low concentrations is known to have reno-protective effects against renal ischemia as a result of its vasodilatory, antioxidant, and anti-inflammatory characteristics. However, due to its very short half-life in circulation, direct measurement of NO is difficult. Instead, for evaluating NO, its metabolites such as nitrite and nitrate, are typically evaluated (57, 58).

IRI in the kidney leads to activation and upregulation of nitric oxide synthase (NOS) (59). Nitric oxide synthase has three isoforms, including endothelial NOS (eNOS), neuronal NOS (nNOS), and inducible NOS (iNOS). eNOS, and nNOS make nitric oxide through short bursts/low concentrations manner that is essential for normal organ functions. However, iNOS, make nitric oxide with high concentrations. The nitric oxide generated by iNOS is toxic, while eNOS serves a protective role (60, 61). Ischemic injury triggers the production of NO in the renal proximal tubules, through iNOS (62), which can be harmful to the kidneys (63). Moreover, ischemia can cause impairment of endothelial function and interfere with the production of eNOS, the endothelial form of NO (64).

#### 3.1.6 Renin-angiotensin system

As significant risk factors during IRI, it can be pointed to RAS activation and angiotensin II (AgII) levels increase (65). AgII contributes to renal injury by constricting renal vessels, increase the sensitivity of vascular system to sympathetic nerve stimulation (66), inducing oxidative stress (67), and promoting apoptosis (68).

RAS has two opposing effects on inflammation in renal tissue mediated by two different pathways: angiotensin-converting enzyme (ACE)/AgII/AT1 receptor, and angiotensin-converting enzyme 2 (ACE2)/(Ag-(1-7))/Mas receptor, which are harmful and protective, respectively (69). During renal ischemia, there is a shift in the balance of the RAS axis (70). ACE2 plays a crucial role in regulating AgII levels by converting AgII to Ag-(1-7), which has many beneficial effects and counteracts the harmful effects of AgII (71).

#### 3.1.7 Complement system

The initiation of IR stimulates complement system (72, 73) which results in the production of biologically active pro-inflammatory agents such as C4a, C3a, C5a, C5b-9, and anaphylatoxins that upregulates adhesion molecules (74). Moreover, C5a and C5b-9 can trigger the expression of selectins and ICAM-1 in endothelial cells (75).

Therapeutic studies have suggested that ischemic preconditioning (IP), a phenomenon where an organ or tissue is exposed to brief ischemia to enhance its adaptability, can be beneficial in the kidney (76). IP can help the kidney tolerate subsequent ischemia-induced injury by inducing a non-lethal period of ischemia (77). IP is believed to reduce cell lysis, apoptosis, lipid peroxidation (78), adhesion molecules, and inflammatory responses according to some therapeutic studies (78, 79). However, other researchers suggest that IP may be mediated through the pre-ischemic activation of adenosine receptors, particularly the A1 adenosine receptors (80).

In summary, renal damage caused by ischemia/reperfusion occurs through a complex interplay of inflammation and various mediators. The production of oxidative stress and lipid peroxidation are significant contributors to the inflammatory process in IRI.

## 4 Noncoding RNAs

**Figure 3:**
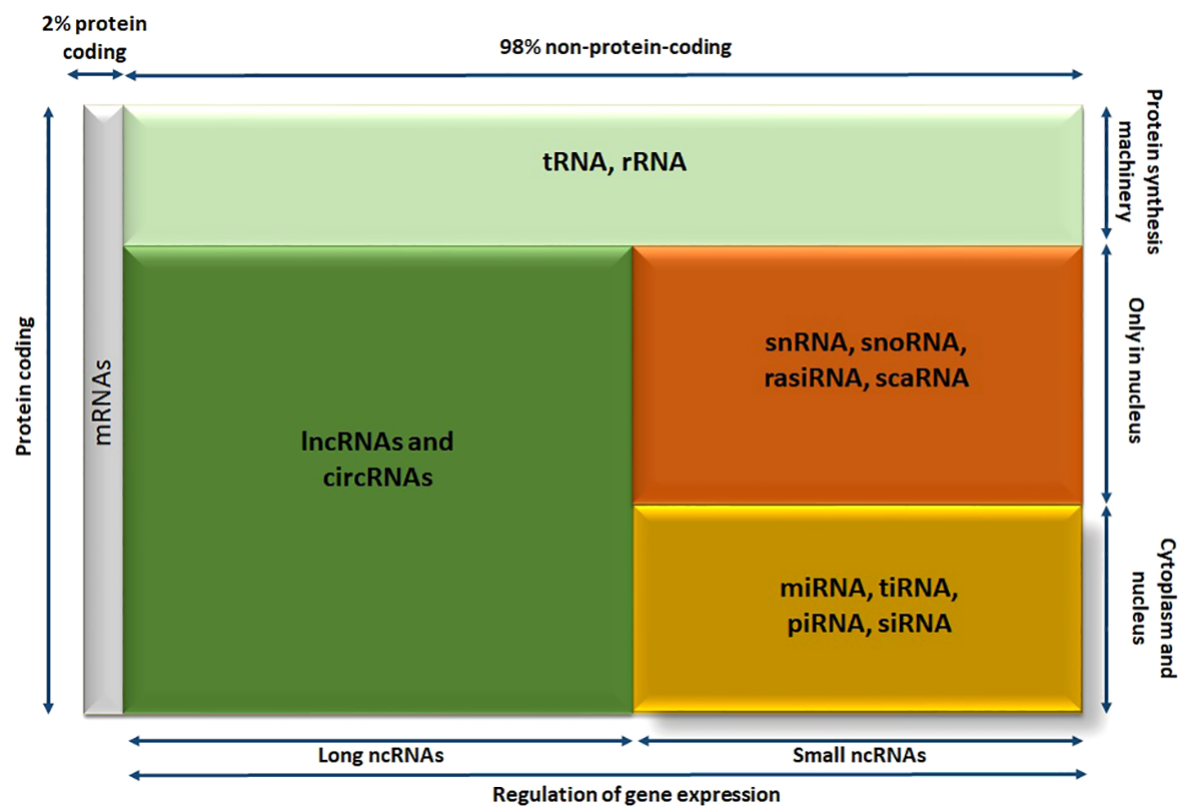
The diagram showing different members of human RNA transcriptome; RNA molecules are divided into various categories according their size, localization, and functionality. circRNA, circular RNA; lncRNA, long noncoding RNA; miRNA, microRNA; piRNA, piwi-interacting RNA; rasiRNA, repeat-associated small interfering RNA; scaRNA, small cajal body-specific RNA; siRNA, small interfering RNA; snoRNA, small nucleolar RNA; snRNA, small nuclear RNA; tiRNA, transfer RNA–derived stress-induced small RNA; tRNA, transfer RNA.

Roughly most of our genes undergo transcription, resulting in the RNA molecules production (**Figure 3**). Consequently, the most parts of the human genome are transcribed into non-protein coding RNAs, commonly referred to as ncRNAs (80%). Except for tRNAs and rRNAs that are the part of protein synthesis machinery, the rest of noncoding RNAs are known to be related in gene regulation processes. The rest is divided into two groups, based on their size called long ncRNAs such as lncRNAs and circRNAs. The second part that is known as small noncoding RNAs are divided into RNA molecules that can only act in nucleus like snRNAs, snoRNAs, rasiRNAs (repeat-associated small interfering RNAs), scaRNAs (small cajal body-specific RNAs) and the RNA molecules that can exist both in cytoplasm and nucleus are comprised of miRNAs, transfer RNA–derived stress-induced small RNAs (tiRNAs), piwi-interacting RNAs (piRNAs), and siRNAs (**Figure 3**) (14).

This family of RNAs is diverse and has multiple functions, with size being a simple criterion for their classification. Small ncRNAs and long ncRNAs are separated by considering 200 nucleotides as an arbitrary cutoff. The category lncRNAs include linear lncRNAs, commonly referred to as lncRNAs, and circRNAs. The same as miRNA molecules, both lncRNAs and circRNAs are regulators of protein coding genes and offer a source of possible new disease markers and therapeutic targets.

Among small ncRNAs, miRNAs are the most extensively studied. These RNA molecules are 20 to 22 nucleotides in length and can decrease the expression level of protein-coding genes, they target. As a result, miRNAs have been suggested as potential biomarkers for various diseases and even as therapeutic targets in the field of personalized medicine (81). Additionally, the members of long ncRNAs meaning, lncRNAs, and circRNAs are studied as having critical function in different pathophysiological processes such as AKI. Therefore, this review will examine the roles of these ncRNA molecules (miRNAs, lncRNAs, and circRNAs) in kidney healthy function and (ischemic) AKI in the subsequent parts (14).

### 4.1 Biogenesis of lncRNAs, miRNAs, and circRNAs

In the following sections the biogenesis of lncRNAs, miRNAs, and circRNAs are reviewed.

#### 4.1.1 Biological features and biogenesis of lncRNAs

The emergence of RNA sequencing methods and bioinformatics strategies has enabled the identification of abundant lncRNAs, which possess several unique biological characteristics. Firstly, they are expressed in large amounts in a wide range of organisms, from prokaryotes to mammals (82). Secondly, they have diverse expression patterns, with a variety of classes originating from different DNA sections in genomes, such as promoters, enhancers, intergenic regions, and the opposite strand of protein-coding genes (about 5,400 to more than 10,000 transcripts in humans). Some of them are also produced through noncanonical RNA processing pathways (83, 84). Thirdly, the expression of lncRNAs is more specific to cell type, tissue, and spatial-temporal localization in comparison to protein-coding genes (85, 86). Fourthly, lncRNA genes are expressed at a minor frequency than protein-coding genes (87). Finally, most lncRNA genes are evolutionarily conserved (84).

lncRNAs are RNA molecules that are transcribed by RNA polymerase II (Pol II) and contain a 5’ methyl-cytosine cap and 3’ poly A tail (88). These molecules are classified into various types based on their unique characteristics. For example, based on their genomic origins, lncRNAs can be categorized into five different classes, including sense, antisense, bidirectional, intronic, and intergenic. Furthermore, based on their role, lncRNAs are classified into three different categories, which are rRNA, tRNA, and cRNA. Moreover, lncRNAs can also be classified based on their subcellular localization into nuclear, cytoplasmic, and mitochondrial lncRNAs (89).

The formation of lncRNAs follows a similar process to mRNA biogenesis, albeit with some alterations in the processes involved. LncRNAs are commonly modified by canonical processes such as capping, polyadenylation, and splicing (90). However, noncanonical processes can also be used, including ribonuclease P (RNase P) cleavage to create mature 3’ ends, capping by snoRNA-protein (snoRNP) complexes, and the production of circular structures (83).

Research suggests that, like protein-coding RNAs, RNA pol II commonly transcribe lncRNAs (91). Nevertheless, some lncRNAs are known to be transcribed by RNA pol III and spRNAP IV (single-polypeptide nuclear RNA polymerase IV) (92). The various types of lncRNAs can be classified as follows.

##### 4.1.1.1 Intergenic and intronic lncRNAs

**Figure 4:**
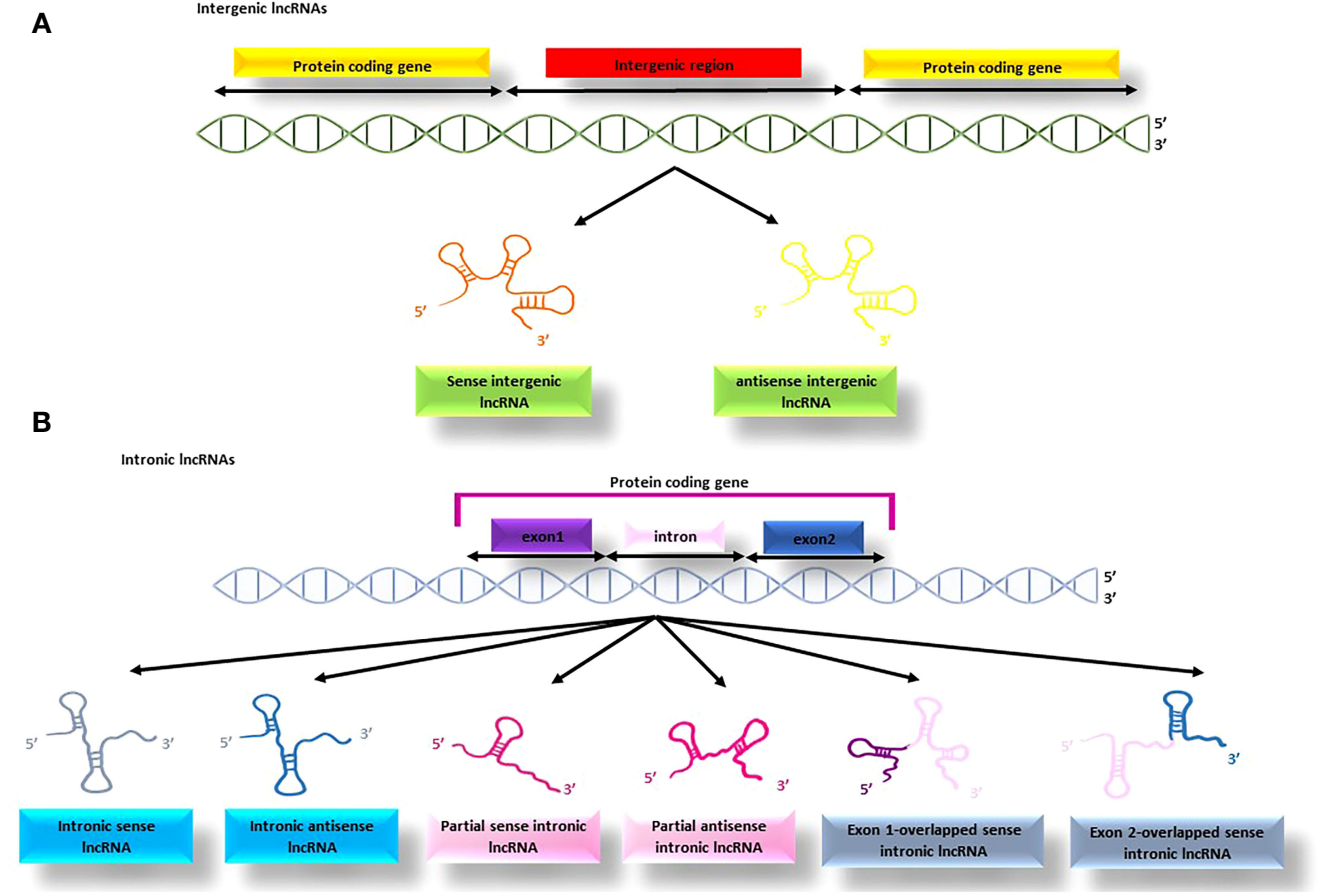
The process of producing intergenic and intronic lncRNAs; **(A)** the lncRNAs produced from the non-coding regions between protein-coding genes are termed intergenic lncRNAs, **(B)** the lncRNAs produced from the protein-coding genes are termed intronic lncRNAs that might be produced from different parts of the gene.

lncRNAs that are transcribed from the non-coding regions between protein-coding genes are called intergenic lncRNAs (as illustrated in **Figure 4A**) (91). The synthesis of lncRNAs are the responsibility of RNA polymerase II or RNA polymerase III and exhibit certain similarities with protein-coding RNAs, such as having a 3’-poly A tail and 5’-capping (93). Additionally, some of these lncRNAs can act as precursors for other types of non-coding RNAs, including microRNAs. For instance, the lncRNA H19 is involved in the production of miR-675 (94).

This type of lncRNA originates from the intronic regions of protein-coding genes and is often produced through alternative splicing, although it may also contain exonic sequences and a 3’ poly A tail. Intronic lncRNAs (see **Figure 4B**) are typically transcribed by RNA polymerase III or spRNAP IV, and some are released from spliced introns to form sno-lncRNAs (95). The binding of sno-lncRNAs to snoRNAs protects them from intronic exonucleolytic end-processing mechanisms (96).

##### 4.1.1.2 Sense (exonic) and antisense lncRNAs

**Figure 5:**
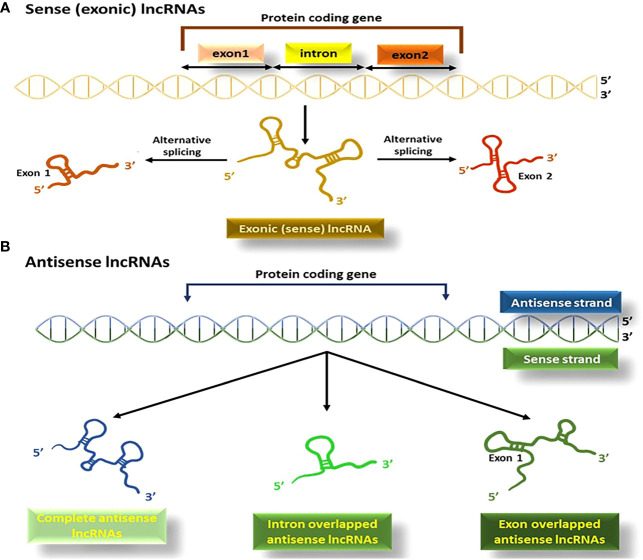
The process of producing sense (exonic) and antisense lncRNAs; **(A)** lncRNAs originating from the exons of the protein coding segment of genes produce exonic lncRNAs, **(B)** Antisense lncRNAs or NATs, are produced in the opposite direction to protein-coding genes.

This type of lncRNAs originates from the sequences of genes that code for proteins and share exons with the corresponding mRNAs (**Figure 5A**). They are typically transcribed by the RNA pol II enzyme and mostly lack functional open reading frames (97). Sense lncRNAs are produced in smaller amounts than their corresponding mRNAs (98).

Antisense lncRNAs (known either as natural antisense transcripts; NATs), are created from the opposite direction to protein-coding genes (99). RNA polymerase III is mainly responsible for their transcription, and they vary in size, consisting of a combination of exons, introns, or the complete sequence of protein-coding genes (**Figure 5B**) (100).

##### 4.1.1.3 Bidirectional and miscellaneous lncRNAs

**Figure 6:**
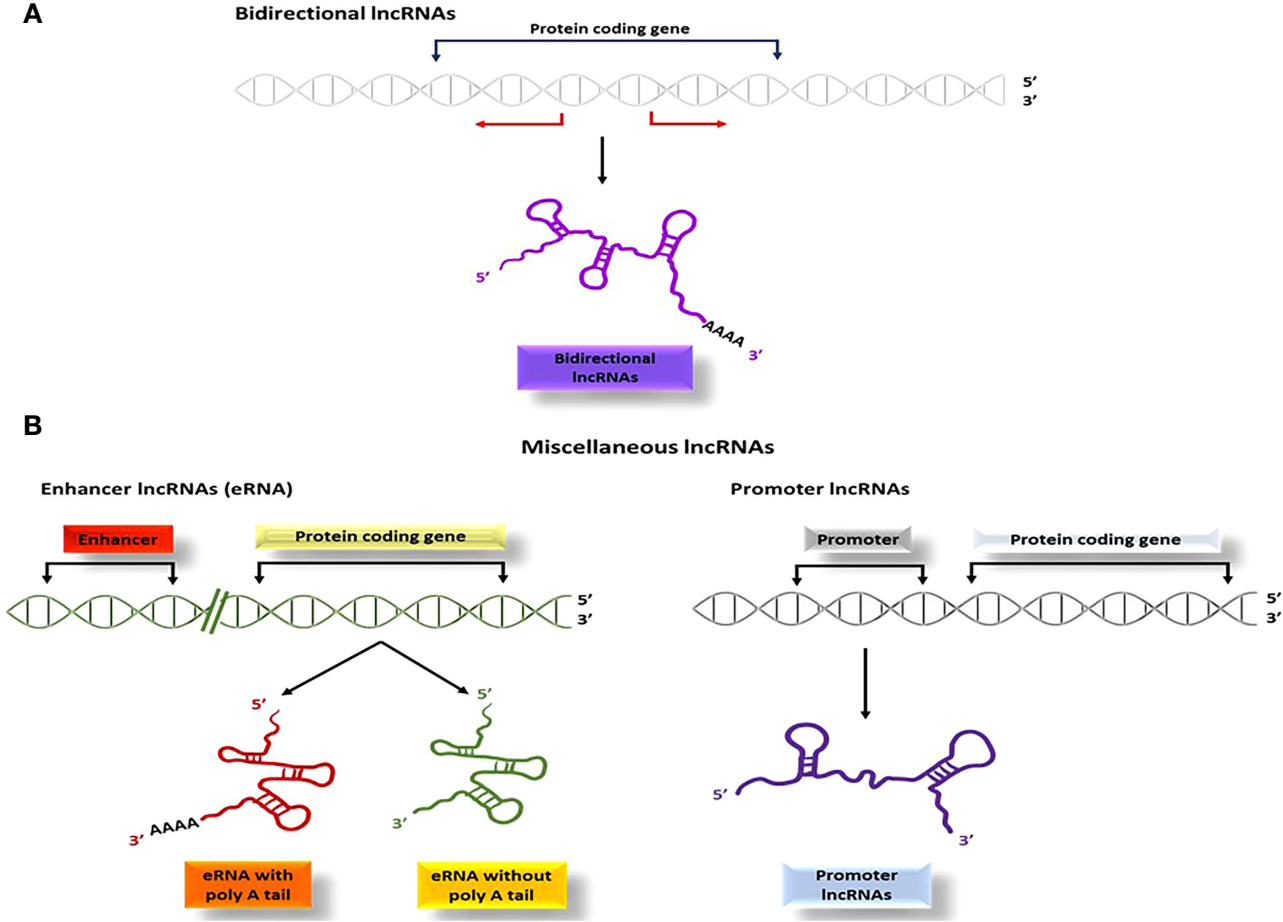
The process of producing bidirectional and miscellaneous lncRNAs; **(A)** bidirectional lncRNAs start transcription within 1000 base pairs to the start sites of the genes and proceed in the opposite direction, **(B)** Several other types of lncRNAs such as pseudo-lncRNAs (resulting from pseudogenes), T-UCR lncRNAs (derived from ultra-conserved regions), enhancer lncRNAs (transcribed from enhancer regions), and promoter lncRNAs (transcribed from promoter sequences), are categorized as miscellaneous lncRNAs.

Bidirectional lncRNAs are a type of lncRNA that start transcription in close vicinity to the protein-coding genes start sites and continue in the opposite direction (within 1000 base pairs) (**Figure 6A**). One noteworthy characteristic of bidirectional lncRNAs is their similar expression pattern to that of their corresponding mRNA partners, which implies that they share regulatory mechanisms and biological functions (101).

Several other categories of lncRNAs are classified due to their site of transcription and is categorized as miscellaneous lncRNAs. Pseudo-lncRNAs arise from pseudogenes (102), T-UCR lncRNAs (transcribed-ultra conserved lncRNAs) are derived from ultra-conserved regions (103, 104), and enhancer lncRNAs or eRNAs (105) are transcribed from enhancer regions via RNA polymerase II or III (**Figure 6B**). Additionally, promoter lncRNAs are generated from promoter sequences (106) (**Figure 6B**).

#### 4.1.2 Biological features and biogenesis of miRNAs

MicroRNAs, a type of small, single-stranded non-coding RNA with a length ranging from 19 to 23 nucleotides, are now known to act as endogenous physiological modulators of gene expression, capable of either inhibiting or promoting the translation or transcription of target mRNAs. The discovery of miRNAs dates back to 1993, when Lee et al. first identified them in Caenorhabditis elegans (107). miRNAs are widely recognized for their role in regulating several physiological and pathological processes like regulating the cell cycle, metabolism, signal transduction, and apoptosis (108, 109).

Primarily the majority of miRNAs are produced through the canonical mechanisms, which has been thoroughly investigated in various research articles [for more detailed information, see (20, 110, 111)]. Within this path, primary miRNAs (pri-miRNAs) are produced from DNA sequences, which are typically composed of 300-1000 nucleotides. Subsequently, the pri-miRNAs undergo additional cleavage at its stem-loop in order to make precursor miRNAs (pre-miRNAs) structures which this reaction is mediated by the RNase III enzyme Drosha (112) along with its cofactor DGCR8 (also known as Pasha) (113), which consists of approximately 70 nucleotides (114).

Subsequently, an exportin 5/RanGTP complex is responsible for translocating the pre-miRNA from the nucleus to the cytoplasm (115). Once in the cytoplasm, additional cleavage by the RNase III enzyme Dicer happens, resulting in the production of a single-stranded mature miRNA (116) that is named by considering its direction, and when originates from the 5’ end of the pre-miRNA hairpin is called 5p, and when originates from the 3’ end of the pre-miRNA hairpin is called 3p. In order to achieve its functional activity, the miRNA enters to an effector complex known as RNA-induced silencing complex (RISC), by interacting with the argonaut (AGO) protein. The RISC complex then attaches to the 3’UTR of its target mRNA(s), resulting in the protein translation repression or mRNA degradation. Notably, the miRNAs bind to a specific region that is called “seed sequence” in order to regulate its target gene(s), leading to either translational repression, mRNA deadenylation, and decapping by 3’UTR binding (117), or silencing by 5’ UTR binding (113).

Generally, the pairing that happens between mammalian miRNAs and its mRNA targets is not entirely complementary (118). When there is perfect complementarity between a miRNA and its target mRNA, the mRNA is mainly cleaved by interfering AGO2 protein (119). However, partial complementarity cause AGO proteins to recruit the GW182 family and facilitate gene silencing (117). In this silencing process, GW182 proteins play a central role, acting as adaptable scaffolds to facilitate the interaction between AGO proteins and downstream effector complexes (120).

Nevertheless, miRNAs have been found to have nuclear functions and can bind to enhancers in the nucleus. Enhancers are genomic cis-regulatory elements that can upregulate gene transcription. Some miRNAs, such as miR-26a-1, miR-339, miR-3179, miR-24-1, and miR-24-2, have been shown to induce the expression of neighboring genes. These miRNAs can activate enhancers and trigger gene transcription in the nucleus (121). Multiple components of the miRNA processing machinery are present in the nuclear compartment, and miRNAs can regulate the stability of nuclear transcripts, induce epigenetic alterations, and modulate alternative splicing events in the nucleus (122). They can also bind to the promoter regions of targeted genes to regulate gene expression in the nucleus (123). Additionally, miRNAs can interact with DNA at sites of active transcription in the nucleus, promoting active or inactive chromatin states (123). Recent studies have also identified a class of miRNAs called NamiRNAs (RNA-nuclear-activating miRNAs) that bind to enhancers in addition to promoters. These NamiRNAs have been found to play a role in the regulation of gene expression in the nucleus (124).

#### 4.1.3 Biological features, biogenesis, and function of circRNAs

Initially in the 1970s, circRNAs were identified in RNA viruses, and were considered as byproducts of mis-splicing, and not very common (125). However, advances in next-generation sequencing (NGS) and bioinformatics analysis, documented that circRNAs are extensively present and possess specific biological functions in the pathogenesis of several diseases (126). Though, circRNAs were initially observed in viruses, they have now been documented to be abundant in eukaryotes, as well as the human transcriptome (127). Moreover, circRNAs are extremely conserved in sequence and expression amongst eukaryotes, making them relatively straightforward to model and investigate (128).

**Figure 7:**
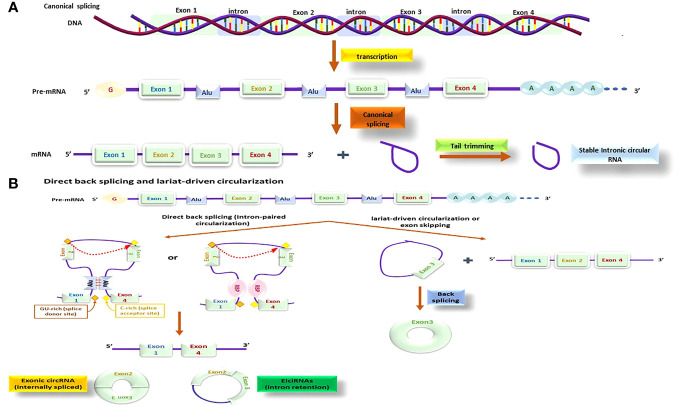
The circRNA production processes; this process might be done by different methods and by using different molecules; beside the canonical process **(A)** there is also other methods that is known as direct back splicing and lariat-driven circularization **(B)** that several other molecules are functioning together to make these processes happen.

Unlike canonical RNA splicing (depicted in **Figure 7A**), circRNAs are typically produced via a process called back splicing which is comprised of fusing a 3’ donor splicing site located in downstream, with an upstream 5’ acceptor site, resulting in a single-strand loop (129). Based on the sequence of splicing procedures and the intermediate products, two biogenesis models have been suggested and confirmed: the direct back-splicing model and the lariat model (illustrated in **Figure 7B**) (130).

The direct back splicing or intron-paired circularization mechanism is facilitated by intronic flanking reverse complementary sequences (ALU repeats), or RNA-binding proteins (RBPs) like quaking (QKI). QKI binds to two different flanking intronic sequences and as a connection brings the 3’ donor into proximity of 5’ acceptor splice sites. Upon splicing, the flanking reverse sequences come together (131) that generates a specific structure called secondary pre-mRNA and splicing starts at the linked exons point, producing a circular RNA. Direct back splicing can produce exonic circRNAs (ecircRNAs) through internal splicing events or exon–intron circRNAs (EIciRNAs) via intron retention. There is also a less studied type of circRNA known as mitochondrial genome-derived circRNAs (132) (**Figure 7B**).

In addition, a distinct type of circRNA is derived from the mitochondrial genome, which has received less attention regarding its functions and roles (133). The lariat-based circularization mechanism, also known as the exon-skipping model, comprises of two splicing events. In the primary step, a nucleophilic attack is started by an adenosine branch point residue at a downstream intron site to GU region of an upstream intron, generating an intron lariat that attaches the upstream exon 3’ end and a downstream exon. Subsequently, the released intron lariat loop promotes RNA circularization (**Figure 7B**) (134).

**Figure 8:**
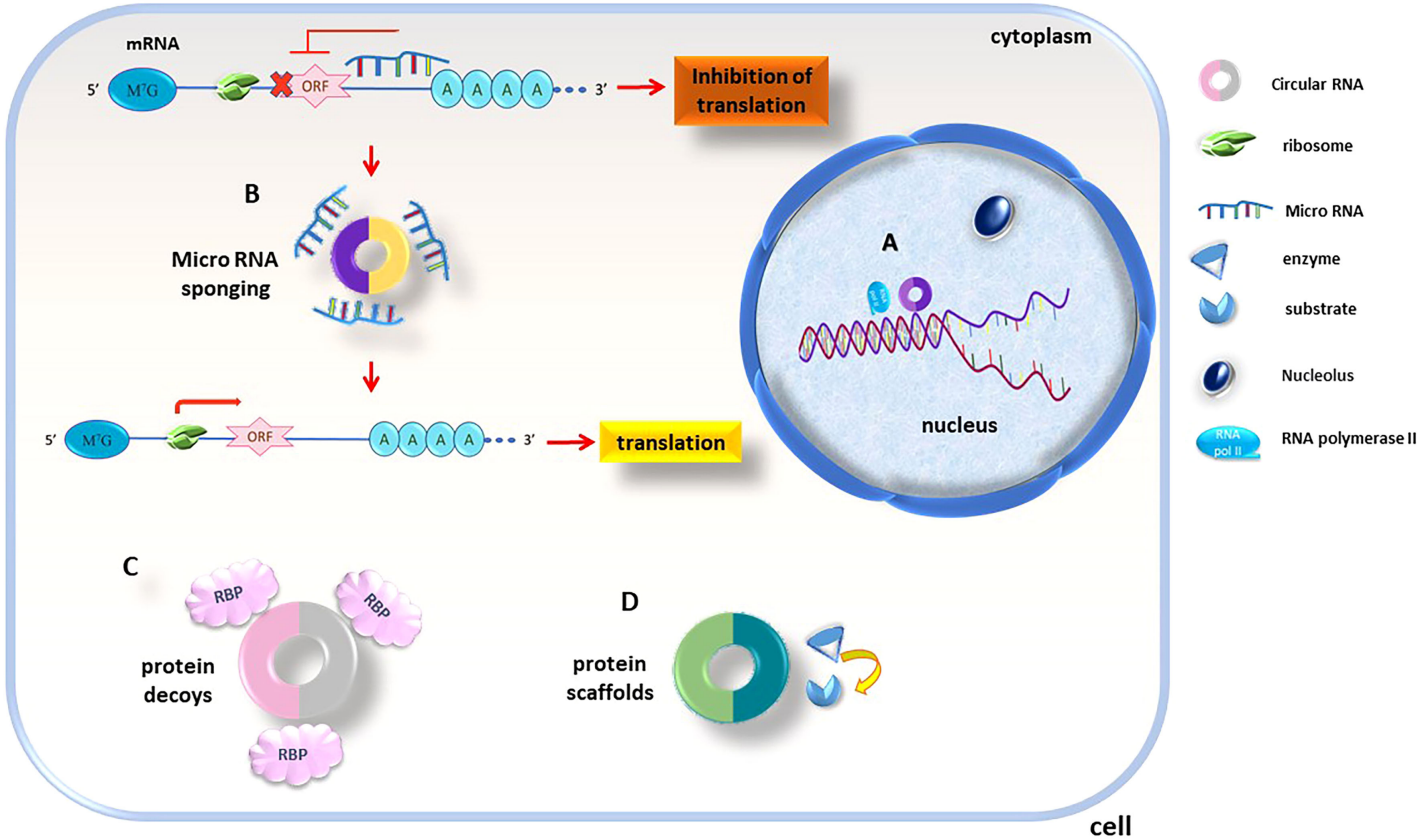
circRNAs functionality is executed thru different mechanisms, circRNAs act as **(A)** transcription regulators and transcription factors and cell cycle checkpoints are found to be targets of circRNA regulation, **(B)** miRNA sponges; as are rich in miRNA-binding sites sponges miRNAs and control their inhibitory effects, **(C)** protein decoys; circRNAs containing RBP binding sequences decoys for these proteins, **(D)** protein scaffolds; regulate intracellular protein/protein and protein/RNA interactions.

According to current knowledge, circRNAs regulate biological pathways through several mechanisms (**Figure 8**). Firstly, they can act as transcription regulators (**Figure 8A**), interacting with regulatory molecules like transcription factors and cell cycle checkpoints. This participation in gene expression regulation happens through epigenetic modification and transcriptional regulation. Secondly, they act as miRNA sponges (**Figure 8B**), regulating target gene expression. circRNAs have abundant miRNA-binding sites and can act as sponges to absorb and regulate miRNA activity, blocking the inhibitory influence of miRNAs on their target genes and thereby, increase the target genes expression level. Thirdly, circRNAs act such as protein decoys (**Figure 8C**). The binding motifs of RBP might act as sponges or decoys and control the function of proteins indirectly. Finally, a few of circRNAs act like protein scaffolds (**Figure 8D**), enabling the enzymes colocalization (e.g., phosphatases, acetylases, and ubiquitin ligases) and their substrates and in this way, insert their impact on the kinetics of subsequent reactions (130).

It is worth mentioning although, circRNAs were initially thought to function as non-coding RNAs, recent evidence has shown that some circRNAs can encode proteins (135, 136) These proteins are synthesized through different mechanism and are typically smaller than canonical proteins (130). The mechanism of protein coding by circRNAs is still an area of active research and is not completely understood. However, several mechanisms have been proposed based on the available evidence:

Rolling circle amplification (RCA) Mechanism: Some studies have suggested that circRNAs with stop codon mutations can utilize a rolling circle amplification mechanism to code for proteins (137).

Cap-independent translation Initiation: this method allows them to bypass the traditional mechanism of translation initiation that requires a 5’ cap structure (135) and can occur through various mechanisms, such as internal ribosome entry sites (IRES) or N6-methyladenosine (m6A) modifications (136).

Interaction with RNA Binding Proteins: circRNAs can interact with RNA binding proteins, which can influence their translation and protein coding potential (130).

Competitive Inhibition of Linear Isoforms: Some circRNA-encoded proteins have been found to competitively inhibit the function of their linearly spliced isoforms (138), by preventing interactions with their binding partners. It is important to note that the mechanisms of circRNA protein coding are still an active area of research, and further studies are needed to fully understand the intricacies of this process.

### 4.2 Roles of lncRNAs in ischemic AKI

Formerly considered “junk DNA”, lncRNAs are now known to perform significant roles in several biological procedures like cell proliferation, apoptosis, and cell cycle progression. They are highly specific and regulated. Many studies (**Table 1**) have shown that lncRNAs are critical for the improvement and progression of AKI, particularly ischemic-induced AKI (165, 166).

**Table 1:** Noncoding RNAs related to ischemic AKI.

ncRNA name	Type	Human	Animal model	Cell line	Target	Process involved	Result	Reference
RP5-1104E15.6-001 (lncRNA-TapSAKI)	lncRNA	AKI biopsy and serum	–	–	Antisense to MGAT3	mortality predictor of AKI patients	Significant increase	(139)
lncRNA-NEAT1	lncRNA	ischemic AKI serum	–	HK‐2 cell injury with CoCl2	miR‐27a‐3p	diagnostic biomarker and therapeutic target in ischemic AKI	Higher expression in ischemic AKI patients	(12)
lncRNA-SNHG14	lncRNA	–	rat	HK‐2 cell	miR-124-3p/MMP2	Knockdown of SNHG14 alleviated IR-induced AKI by miR-124-3p-mediated downregulation of MMP2	Increased expression in IR and HR models	(140)
ncRNA-Malat1	lncRNA	biopsies and plasma of AKI patients	–	HUVECs and HK-2 cells by HR	–	upregulated in renal IR injury thru HIF-1α	upregulated in renal IR injury	(141)
lncRNA-TUG1	lncRNA	–	IR-induced mice	HR-induced HK-2	miR-494-3p/E-cadherin	reduced both in IR-induced mice and HR-induced HK-2 cells	might regulate HR-induced cell injury through miR-494-3p/E-cadherin axis	(142)
miR-687	miRNA	–	mice	HR-induced HEK	PTEN	HIF-1/miR-687/PTEN signaling pathway in IRI	upregulated in renal IR	(143)
miR-494	miRNA	patients with AKI (urine)	–	–	ATF3	binds to ATF3 and decreases its transcription, in AKI patients	a novel renal tubular cell injury biomarker for early AKI	(144)
miR-24	miRNA	Kidney transplantation induced AKI	mice	Anoxia/hypoxia induced in umbilical vein endothelial cells and HK-2	H2A.X and HO-1 and S1PR1	miR-24 promotes renal ischemic injury by stimulating apoptosis in endothelial and tubular epithelial cell	upregulated in the kidney after IRI	(145)
miR-16	miRNA	AKI patients’ plasma	–	–	–	may serve as a novel biomarker AKI	Down-regulated in the plasma of kidney injury AKI patients	(146)
miR-320	miRNA	AKI patients’ plasma	–	–	–	may serve as a novel biomarker AKI	Down-regulated in the plasma of kidney injury AKI patients	(146)
miR-210	miRNA	AKI patients’ plasma	–	–	–	may serve as a novel biomarker AKI	up-regulated in the plasma of kidney injury AKI patients	(146)
miR-30c-5p	miRNA	CSA-AKI patients’ urine	IR-induced rat urine	Hypoxia induced HK-2 cells	–	elevated significantly in rat model and AKI patients	IR-induced AKI potential diagnostic marker	(147)
miR-192-5p	miRNA	CSA-AKI patients’ urine	IR-induced rat urine	Hypoxia induced HK-2 cells	–	elevated significantly in rat model and AKI patients	IR-induced AKI potential diagnostic marker	(147)
miR-378a-3p	miRNA	patients’ urine with cardiac surgery	IR-induced rat urine	Hypoxia induced HK-2 cells	–	elevated significantly post operation	More important marker in rat model	(147)
miR-192-5p	miRNA	CSA-AKI patients	plasma and kidney tissue of IRI rats	–	–	4x increase in plasma and 40% decrease in IRI rat kidney/increase in ICU time	potential plasma marker for AKI	(148)
miR-486-5p	miRNA	–	IR-induced mice	–	PTEN and the Akt pathway	miR-486-5p targeting PTEN reduces IRI effects	miR-486-5p enriched exosomes could represent a therapeutic tool in AKI	(149)
miR-21	miRNA	–	IR-induced C57BL/6 mice	IR-induced C57BL/6 mice TEC isolated	–	Act in both IRI induced cell-intrinsic and -extrinsic mechanisms	up-regulated in IRI mechanisms/miR-21 knockdown in TEC increased cell death	(150)
miR-21	miRNA	–	IR-induced mice	IR-induced BMDCs	CCR7	IRI suppressed miR-21 expression in BMDCs	miR-21transfer to BMDCs alleviate AKI	(151)
miR-21	miRNA	urine and plasma of CSA-AKI patients	–	–	–	miR-21 in urine and plasma deals with severe AKI induced by cardiac surgery	miR-21 up-regulation in urine and plasma relates with AKI progression	(152)
miR-21	miRNA	urine and serum of CSA-AKI patients	–	–	–	urinary miR-21 is a more reliable marker for detection of CSA-AKI	miR-21 decreased expression in CSA-AKI patients	(153)
miR-101-3p, miR-127-3p, miR-210-3p, miR-126-3p, miR-26b-5p, miR-29a-3p, miR-146a-5p, miR-27a-3p, miR-93-3p, miR-10a-5p	miRNA	Serum sample of AKI patients	–	–	–	AKI diagnosis in ICU patients	set of increased serum miRNAs as AKI biomarkers for early detection with high diagnostic value	(154)
miR-21, miR-200c, miR-423, and miR-4640	miRNA	Urine sample of AKI patients	–	–	–	panel of miRNAs as sensitive indicators of kidney damage	Increase in urine of AKI patients	(155)
ciRs-126	circRNA	blood samples of AKI patients	–	–	miR-126	ciRs-126 sponge miR-126-5p, which was highly suppressed in AKI patients and hypoxic endothelial cells	highly increased in blood of AKI patients	(13)
ciRs-YAP1	circRNA	AKI blood samples	–	IR-treated HK-2 cells	miR-21-5p	circYAP1 activated PI3K/AKT/mTOR pathway via inhibiting miR-21-5p	Decrease in blood of AKI patients	(156)
circ-ITGB1	circRNA	–	–	HR−exposed HK−2	miR-328-3p/PIM1	HR−induced inflammatory enhance circITGB1 which response via miR−328−3p/PIM1 axis	Increase during HR induction	(157)
circ-ZNF609	circRNA	AKI in heart disease patients’ urine	–	HK-2 cells	ZNF609-250aa/AKT3/mTOR signaling	encodes a ZNF609-250aa which activate AKT3/mTOR signaling and induce autophagy flux impairment and cell apoptosis	highly expressed in the kidney after IRI	(158)
circBNC2	circRNA	human IRI-induced chronic kidney disease	–	–	G2/M	Decreased expression of circBNC2 is related to increased G2/M arrest	significantly downregulated after acute ischemic condition	(159)
circ-0005519	circRNA	asphyxia-induced AKI	–	–	Targeting hsa-let-7a-5p and production of IL-13 and IL-6	participate in inflammation response in AKI development	significantly upregulated	(160)
circ-Dnmt3a, circ-Akt3, circ-Plekha7, and circ-Me1	circRNA	–	Ischemic AKI rat models	–	PI3K-Akt signaling pathway	participate in inflammation response in AKI rat model	down-regulated in AKI rats	(161)
circ-0114427	circRNA	–	cisplatin-induced AKI mice model renal tubular tissues	AKI cell model	miR-494/ATF3/IL-6 pathway	Acts via miR-494/ATF3/IL-6 pathway to regulate inflammation during AKI	significantly up-regulated in different AKI cell models	(162)
circRNA-45478	circRNA	–	C57BL/6 mice kidneys	BUMPT cells	miR-190a-5p/PHLPP1	mediated IR-induced apoptosis	increase in ischemic AKI via miR-190a-5p/PHLPP1 pathway	(163)
circ-Snrk	circRNA	–	AKI rat model	HR cell model (NRK-52E cells)	MAPK signaling	involved in AKI development thru MAPK signaling	increase in AKI via MAPK signaling	(164)

AKI, Acute kidney injury; HR, hypoxia/reoxygenation; HIF-1α, Hypoxia-inducible Factor-1α; IRI, Ischemic/reperfusion injury; PTEN, phosphatase and tensin homology; ATF3, activating transcription factor 3; H2A.X, H2A histone family, member X; HO-1, heme oxygenase-1; S1PR1, sphingosine-1-phosphate receptor 1; PDCD4, programmed cell death protein 4; TEC, tubular epithelial cells; DC, dendritic cells; CCR7, chemokine C receptor 7; BMDCs, bone marrow-derived DCs; CSA-AKI, cardiac surgery associated AKI; PIM1, pim−1 proto−oncogene; BUMPT cells, mouse proximal tubule-derived cell lines; PHLPP1, Pleckstrin homology (PH) domain leucine-rich repeat protein phosphatase 1.

RP5-1104E15.6-001, also known as TapSAKI (Transcript Predicting Survival in AKI), is an intronic lncRNA originating from the RP5-1104E15.6 gene (located on chromosome 22) that runs antisense to β-1,4-mannosyl-glycoprotein 4-β-N-acetyl glucosaminyl transferase (MGAT3). TapSAKI is detectable in studied kidney biopsies and upregulated in the AKI patients’ plasma. Analysis showed TapSAKI to be an independent predictor of 28-day survival and enriched in tubular epithelial cells exposed to ATP depletion. The altered amounts of circulating lncRNAs in AKI patients support the TapSAKI importance as a mortality predictor agent (139).

Another study investigated the expression and role of the lncRNA nuclear enriched abundant transcript 1 (NEAT1) in serum samples from patients with ischemic AKI and *in vitro* models. The study outcomes exhibited a substantial increase in NEAT1 expression in patients harboring ischemic AKI and that inhibiting NEAT1 expression reduced CoCl2-induced injury in HK-2 cells. NEAT1 was detected to negatively regulate miR-27a-3p expression, suggesting it might be a promising diagnostic biomarker and therapeutic target for ischemic AKI (12).

AKI models created both in *in vivo* and *in vitro* were created by applying IR and hypoxia/reoxygenation (HR) to rats and HK-2 cells. The of Small nucleolar RNA host gene 14 (SNHG14) was expressed increasingly, while miR-124-3p was decreased in HK-2 cells subjected to HR. SNHG14 targeted miR-124-3p, and miR-124-3p targeted MMP2. Suppressing miR-124-3p or overexpression of MMP2 reversed the inhibitory effects of SNHG14 on inflammation and oxidative stress and restored the stimulating effect of SNHG14 on cell sustainability in HK-2 cells subjected to HR. These findings propose that SNHG14, miR-124-3p, and MMP2 possibly will play important roles in the pathogenesis of AKI (140).

The hypoxia-induced lncRNA recognized as Metastasis Associated Lung Adenocarcinoma Transcript 1 (Malat1) is upregulated in renal IRI. Malat1 expression was diagnosed to be increased in kidney biopsies and plasma samples of AKI patients. The transcriptional activation of Malat1 was observed to be mediated by hypoxia-inducible factor (HIF)1-α, which is consistent with the induction of Malat1 in cultured cells (HUVECs and HK-2 cells) under HR conditions. It was also found that HIF-1α plays a role in the transcriptional activation of Malat1 in HUVEC and HK-2 cells (141).

The study investigated the potential of lncRNA-TUG1 in alleviating AKI induced by IR. It was observed that lncRNA-TUG1 expression was down-regulated both in mice with IR-induced AKI and HK-2 cells subjected to HR. Additionally, when TUG1 was overexpressed, it had a significant reducing effect on apoptosis and inflammatory cytokines in HR-induced HK-2 cells. Moreover, TUG1 negatively regulate miR-494-3p, which was found in high levels in IR-induced AKI. In this manner, TUG1 suppresses the ATF3 gene activity that act as kidney protection. All of these processes result in apoptosis initiation and aggravation of kidney injury. Therefore, E-cadherin was selected as a miR-494-3p target. These findings suggest that TUG1 has the potential to be a therapeutic target for AKI induced by IR (142, 144). The effects of TUG1 up-regulation were reversed when miR-494-3p mimics were used by stimulating the production of IL-1β, TNF-α, and IL-6, which led to cell apoptosis and up-regulation of Bax and cleaved-caspase3, while down-regulating Bcl-2. Additionally, the protein level of E-cadherin was down-regulated by the miR-494-3p mimics (167, 168).

Additionally, ferroptosis was discovered as a new regulator of cell death which is dependent on iron and ROS (169). Mis-regulated ferroptosis has been demonstrated in multiple physiological and pathological processes like cancer, tissue injury, T cell immunity (170). In the recent study by Sun et al., it was showed that lncRNA TUG1 carried by human urine-derived stem cells-derived exosomes may regulate Acyl-CoA synthetase long-chain family member 4-mediated cell ferroptosis by interacting with Serine/arginine splicing factor 1 and then alleviate IRI-AKI (171).

### 4.3 Roles of miRNAs in ischemic AKI

In recent years, increasing evidence has supported the involvement of miRNAs in the pathogenesis of various renal diseases (172) (**Table 1**). Therefore, improving functional analysis approaches for miRNAs in AKI can provide valuable information on their roles in various cellular mechanisms and identify abnormal expression patterns under these conditions. In the study by Wei et al., they created a conditional knockout mouse model by specific removing of Dicer enzyme from kidney proximal tubule (PT) cells, provided the initial proof of miRNAs’ pathological functions in AKI condition. In this model, more than 80% of miRNAs were depleted from the renal cortex, and the PT-Dicer-/- mice demonstrated notable resistance to ischemic AKI by exhibiting considerable improved renal function, minor tissue injury, less apoptosis in tubular cells, and higher survival rates following IR condition (173). These outcomes propose that Dicer and the associated production of miRNA are critical in the development of ischemic AKI (20).

Lorenzen et al. analyzed the miRNA profile of patients with AKI and healthy controls. They validated their findings in AKI patients, healthy controls, and critically ill patients suffering from acute myocardial infarction. The results revealed that in patients with AKI, the plasma levels of miR-16 and miR-320 were reduced, while miR-210 was increased compared to healthy and disease controls. The upregulation of miR-210 may indicate mortality and could be used as an innovative biomarker for AKI that shows alterations on a cellular level (146). In another study, miR-687, which acts as a critical regulator and therapeutic target in renal IRI, is markedly upregulated in the kidneys of mice with renal IR injury and in HR-induced HEK cells, a process mediated by HIF-1. By repressing the expression of phosphatase and tensin homolog (PTEN), miR-687 promotes cell cycle progression and apoptosis. These results have revealed a novel HIF-1/miR-687/PTEN signaling pathway involved in IRI. Further studies on the promoter sequence of miR-687 discovered a putative hypoxia-responsive element (HRE) or HIF-binding site, proposing that HIF may directly regulate the transcription of miR-687 (143).

In patients with AKI, urinary miR-494 has been found to attach to the 3’UTR of ATF3 and decrease its transcription (174). Interestingly, the serum levels of miR-494 showed no significant changes in healthy controls, ICU patients without AKI, and AKI ICU patients. The urinary levels of miR-494 were meaningfully greater in AKI ICU patients than in the normal control group (144). Initially identified as an upregulated miRNA in human retinoblastoma tissues and Waldenström macroglobulinemia cells (175, 176), miR-494 has been found to be increased in various conditions such as myocardial and microvascular endothelial cells in type 2 diabetic rats (177) or ex vivo IR mouse hearts (178). On the other hand, miR-494 was reduced in head and neck squamous cell carcinoma primary tissues (179). miR-494 overexpression, stimulate the production of some inflammatory mediators (P-selectin, MCP-1, and IL-6), and also promote the inflammatory immune responses which are NF-kB-dependent. The result of these immunologic procedures is increase in apoptosis and further decrease in renal function (144). According to a study on AKI in mice, it was observed that the ATF3 mRNA level significantly increased in urinary exosomes and kidney tissue as early as one hour after IR (180). This finding was reported in a review article on miRNA and AKI. In a previous study by Yoshida et al., it was shown that overexpression of ATF3 in mice improved IRI, while ATF3 knockout mice had higher mortality and kidney dysfunction due to renal IR (181).

MiR-24 was found to be upregulated in the kidney after IRI in mice and patients, as well as in anoxia/hypoxia-induced human umbilical vein endothelial cells and tubular epithelial cells (HK-2). Studies proved that a transient increase in the expression level of miR-24 can induce apoptosis in endothelial and tubular epithelial cells, while silencing miR-24 reduce the apoptosis and improved tissue function during hypoxic situations. Downregulation of sphingosine-1-phosphate receptor 1 (S1PR1), H2A histone family member X (H2A.X), and heme oxygenase-1 (HO-1) protein, that were certified to be targets of miR-24 via luciferase reporter assays, was responsible for enhanced apoptosis in proximal tubular and endothelial cells during miR-24 overexpression conditions (145). H2A.X and HO-1 are known to protect cell from DNA damage and oxidative stress (182, 183), while S1P1R agonists activate cell viability by activating the Akt and/or mitogen-activated protein kinase (MAPK) pathways and directly block apoptosis (184).

In a rat model of IR-induced kidney injury, miR-30c-5p, miR-192-5p, and miR-378a-3p expression level was significantly increased in the urine post-operation. In AKI patients, elevated levels of miR-30c-5p and miR-192-5p were observed 2 hours post-operation, and miR-30c-5p displayed better diagnostic value comparing to current protein markers (147).

In 2017, a study examined the expression of miR-192 in both plasma and kidney tissue samples of IRI-induced rats at various time points after stimulation of kidney injury. The findings were then confirmed in the plasma of studied cardiac surgery induced AKI patients. A miRNA profiling analysis identified 42 miRNAs that were found to be expressed differentially in the IRI rat models comparing to the sham group, and based on these findings, miR-192 was selected for further validation since it was enriched in the kidney. Further analysis of miR-192 expression, using Real-time PCR method, verified the plasma fourfold increase and near 40% decrease in kidney samples of IRI rats. In patients with AKI, the plasma miR-192 level increased upon admission to the ICU, remained stable for two hours, and then declined after 24 hours (148).

Although miR-21 is consistently associated with AKI, its role is complex and both knockdown and overexpression do not lead to adverse effects (185). In a study conducted on C57BL/6 mice with ischemia-induced kidney injury, global miRNA expression profiling identified some differentially expressed miRNAs (miR-21, miR-20a, miR-146a, miR-199a-3p, miR-214, miR-192, miR-187, miR-805, and miR-194). These changes were observed to be independent of lymphocyte infiltration, suggesting a lymphocyte-independent signature of renal IRI. *In vitro* investigations certified the important role of miR-21 in proliferation and up-regulation of tubular epithelial cells (TECs) through cell-intrinsic (resulted from ischemia) and -extrinsic (resulted from TGF-β signaling) mechanisms. Further studies in TECs revealed that miR-21 knock-downing strategies increased cell death, while increased expression of this miRNA inhibits cell death. Nevertheless, after ischemia, miR-21 overexpression alone was insufficient for inhibiting TECs from death. These results provide insight into the molecular fingerprint of renal injury and suggest a potential role of miR-21 in protecting TEC from death (150).

Induction of HR decreased miR-21 expression in bone marrow-derived dendritic cells (BMDCs) and increased the percentage of mature DCs, in mice. On the other hand, miR-21 mimics induced anti-inflammatory DC response and immature DC phenotype. Transferring BMDCs with miR-21-overexpression could alleviate renal IR and AKI. In this study, chemokine C receptor 7 (CCR7) also identified to be miR-21 target gene, therefore, CCR7 silencing in BMDCs decrease mature DCs under HR (151).

In an investigation conducted on patients undergoing cardiac surgery, the levels of miR-21 increased in both urine and plasma that linked to the progression of AKI. The findings suggested that urinary and plasma miR-21 levels could serve as prognostic markers for severe AKI and other negative postoperative outcomes in cardiac surgery patients (152).

A study was conducted to investigate whether miR-21 could detect CSA-AKI earlier than serum Cr. The levels of miR-21 in serum and urine were measured before and after surgery at 6, 12, and 24 hours. The results indicate that both postoperative serum and urinary miR-21 levels can predict the development of AKI, but urinary miR-21, especially at 6 hours after surgery, is a more reliable marker for diagnosing established CSA-AKI compared to serum miR-21 (153). These findings suggest that miR-21 has multiple targets with a complex regulatory network.

The study utilized qRT-PCR arrays on serum samples to demonstrate that 10 specific miRNAs (miR-101-3p, miR-127-3p, miR-210-3p, miR-126-3p, miR-26b-5p, miR-29a-3p, miR-146a-5p, miR-27a-3p, miR-93-3p and miR-10a-5p) can serve as diagnostic biomarkers for AKI in ICU patients with nearly 100% specificity and sensitivity. These findings suggest that this panel of serum miRNAs can be utilized as an effective tool for early detection of AKI and possess high diagnostic value (154). The study also found that four specific miRNAs (miR-26b-5p, miR-146a-5p, miR-93-3p, and miR-127-3p) showed a gradual decrease in their serum levels leading up to the diagnosis of AKI by serum Cr after cardiac surgery (154).

After analyzing the urinary miRNA levels, it was discovered that four miRNAs (miR-21, miR-200c, miR-423, and miR-4640) could effectively discriminate AKI patients from others. When the results of these four miRNAs were combined, the area under the ROC curve was 0.91, indicating a high level of accuracy (155).

A study on mice animal model elucidated that human cord blood endothelial colony-forming cells (ECFCs) or their exosomes can protect from kidney IRI. The exosomes from ECFCs were found to have high amounts of miR-486-5p, that targets PTEN and the Akt pathway. Therefore, treating hypoxic endothelial cells with ECFCs conditioned medium that were supplemented with anti-miR-486-5p, blocked the increase in miR-486-5p and phosphorylated Akt, restored PTEN expression, and enhanced apoptosis. Thus, exosomes enriched with miR-486-5p could serve as a therapeutic tool for treating AKI (149).

### 4.4 Roles of circRNAs in ischemic AKI

Due to their stable structure and specificity to tissues, circRNAs hold significant potential in the early diagnosis and treatment of AKI by altering the urine and blood contents. However, more research should be executed in order to detect specifically expressed circRNAs during the AKI development, and further basic studies are required to evaluate their functions (**Table 1**). In this regard, circRNA analysis has been widely employed in the study of kidney disease. An RNA sequencing study revealed that 1664 circRNAs are considerably expressed in human kidney tissues, of which 474 are exclusive to the kidney (186).

Previously, it was observed that circTLK1 is involved in sepsis-induced AKI by controlling inflammation and oxidative stress via the miR-106a-5p/HMGB1 axis. Several studies have suggested that targeting circTLK1/miR-106a-5p may be a promising approach for AKI treatment (187). Additionally, a comprehensive analysis of global circRNA expression in blood samples from AKI patients revealed that the circular RNA sponge of miR-126 (ciRs-126) was significantly elevated in comparison to healthy and disease controls. Bioinformatic analysis indicated that ciRs-126 sponges miR-126-5p, which is highly suppressed in AKI patients and hypoxic endothelial cells. Therefore, ciRs-126 may serve as a potential prognostic marker for AKI-related mortality (13).

CircYAP1 was found to be down-regulated in blood samples of AKI patients and in HK-2 cells treated with IR. Overexpression of circYAP1 promoted cell growth, reduced secretion of inflammatory factors, and decreased the production of ROS in IR-treated cells. Additionally, circYAP1 was found to act as a sponge for miR-21-5p. Interestingly, overexpression of miR-21-5p reversed the inhibitory effects of circYAP1 on cell injury. Furthermore, circYAP1 activated the PI3K/AKT/mTOR pathway by inhibiting miR-21-5p (156).

The downregulation of miR-328-3p was observed in HK-2 cells exposed to HR, which was attributed to the overexpression of circular RNA integrin beta 1 (circITGB1). The target of miR-328-3p is the Pim-1 proto-oncogene (PIM1), and its overexpression led to an increase in cell viability and a decrease in apoptosis, as well as a decrease in the expression of inflammatory factors following HR exposure. In addition, the functional ITGB1 promoter sequence contains a binding site for the transcription factor GATA binding protein 1 (GATA1), which promotes the expression of circITGB1(157).

The expression of urinary circ-ZNF609 was significantly elevated in the kidneys of AKI-heart disease patients after IRI, resulting in the activation of AKT3/mTOR signaling, which in turn leads to autophagy flux impairment and cell apoptosis *in vitro* in HK-2 cells and *in vivo* in AKI kidneys (158).

The circRNA circBNC2, which is present in high levels in normal renal tubular cells and hepatocytes, experiences significant downregulation after acute ischemic or toxic injury. The reduced expression of circBNC2, as well as an increase in G2/M arrest of epithelial cells, is also observed in patients with CKD induced by IRI, emphasizing its clinical relevance (159).

A study revealed that in neonates with asphyxia-induced AKI, circ-0008898 and circ-0005519 were significantly upregulated, whereas circ-0132279, circ-0017647, and circ-0112327 were down-regulated compared to the control group. Among these circRNAs, circ-0005519 (which is situated in the SNX13 gene) may participate in the inflammation response during AKI development by modulating T-cell differentiation, apoptosis, and cytokine secretion. This is achieved by targeting hsa-let-7a-5p, which promotes the expression of IL-13 and IL-6 (160, 188, 189).

The investigation showed that circ-Dnmt3a, circ-Akt3, circ-Plekha7, and circ-Me1 were downregulated in rats with AKI and then restored by the use of losartan via the PI3K-Akt signaling pathway. The study provides a comprehensive view of the expression patterns of circRNAs by examining the suppressive effects of losartan on AKI caused by ischemia in rats (161).

The circRNA content of mouse renal tubular tissues was observed in a cisplatin-induced AKI model, and circ-0114427 was identified and further studied for its role in an AKI cell model. circ-0114427 expression significantly increased in various AKI cell models, and it was shown to regulate the inflammatory response in the early stages of AKI through the circ-0114427/miR-494/ATF3/IL-6 pathway (162).

A study demonstrated the involvement of circRNA-45478 in ischemic injury-induced apoptosis in mouse proximal tubule-derived cell lines (BUMPT cells) and C57BL/6 mouse kidneys. circRNA-45478 facilitated apoptosis in BUMPT cells under IR conditions by acting as a miR-190a-5p sponge and subsequently upregulating Pleckstrin homology (PH) domain leucine-rich repeat protein phosphatase 1 (PHLPP1). In conclusion, the circRNA-45478/miR-190a-5p/PHLPP1 axis has a central part in the ischemic AKI development (163).

A study utilized an AKI rat model and NRK-52E cell model to examine the function of circ-Snrk. Results exhibited that circ-Snrk knockdown situation renders in NRK-52E cells apoptosis suppression, reduced secretion of inflammatory cytokines (IL-6 and TNF-a), and inhibited the activation of p-JNK and p-38 transcription factors. circ-Snrk expression was found to be upregulated during AKI and was associated with the development of AKI through the MAPK signaling pathway. (164).

## 5 Future perspectives and probable clinical applications of noncoding RNAs in ischemic AKI

lncRNAs, miRNAs, and circRNAs are potential candidates to act as biomarkers or therapeutic targets of AKI (14); however, further preclinical and clinical trials are necessary to confirm their efficacy. In terms of therapeutic strategies, modulation of the complement system has shown promise in mitigating IRI. For example, specific C5a receptor antagonists have demonstrated protective effects against renal IRI, while interfering RNA (siRNA) targeting C3 and caspase 3 genes have reduced renal IRI (190).

Studies have shown that miRNAs possess significant potential in both the diagnosis and treatment of a range of diseases. In particular, their value as diagnostic biomarkers stem from their remarkable stability in bodily fluids, such as plasma, as well as their detectability, which surpasses that of plasma proteins. This has been highlighted in a study (191), where it was reported that miRNAs are readily detectable in fluids and hold promise as diagnostic tools (14).

Several significant biological networks, including cell proliferation (MAPK pathway), apoptosis (PI3K/Akt pathway), and inflammation (NF-κB pathway), have been identified as the mechanisms through which circRNAs operate in diverse sorts of tissue injuries. Though, as a result of the intricate nature of intracellular signaling pathways, still, the only ascertained circRNAs interactions have been introduced within a single mechanism axis. Considering their function as miRNA sponges, protein decoys, and transcription regulators, circRNAs might have different biological roles that still need more investigations to be entirely detected. Consequently, the circRNAs precise therapeutic ability remains blurred until we gain a more profound understanding of the underlying intracellular interactions (192) and their feasibility as therapeutic targets (192). It should not be dismissed that the relatively long half-life of circRNAs due to their covalently enclosed-loop structure, potentiate them as powerful therapeutic agents (193). Additionally, circRNAs are exceedingly abundant and display significant dysregulated expression profiles and act in a pathology-dependent manner in organ injuries with similar pathologies (194).

However, the present studies have several limitations that need to be addressed. Firstly, the majority of these studies primarily relied on human cell and animal models, with limited examination of patient-derived samples. Hence, further investigations should focus on studying the interactions among multiple species of circRNAs within a microenvironment that closely mimics the intracellular conditions specific to a particular disease pathology during organ injury. Secondly, the existing studies only scratch the surface in understanding the function of individual circRNAs. To gain a comprehensive understanding, it is necessary to explore the complex interplay between various circRNA species. Lastly, due to the growing interest in this research field, an increasing number of circRNAs are being reported and documented in extensive databases. Therefore, to effectively assess the practical applicability of circRNAs in clinical settings, further studies should be done for producing standard protocols in this area of research (195).

**Figure 9:**
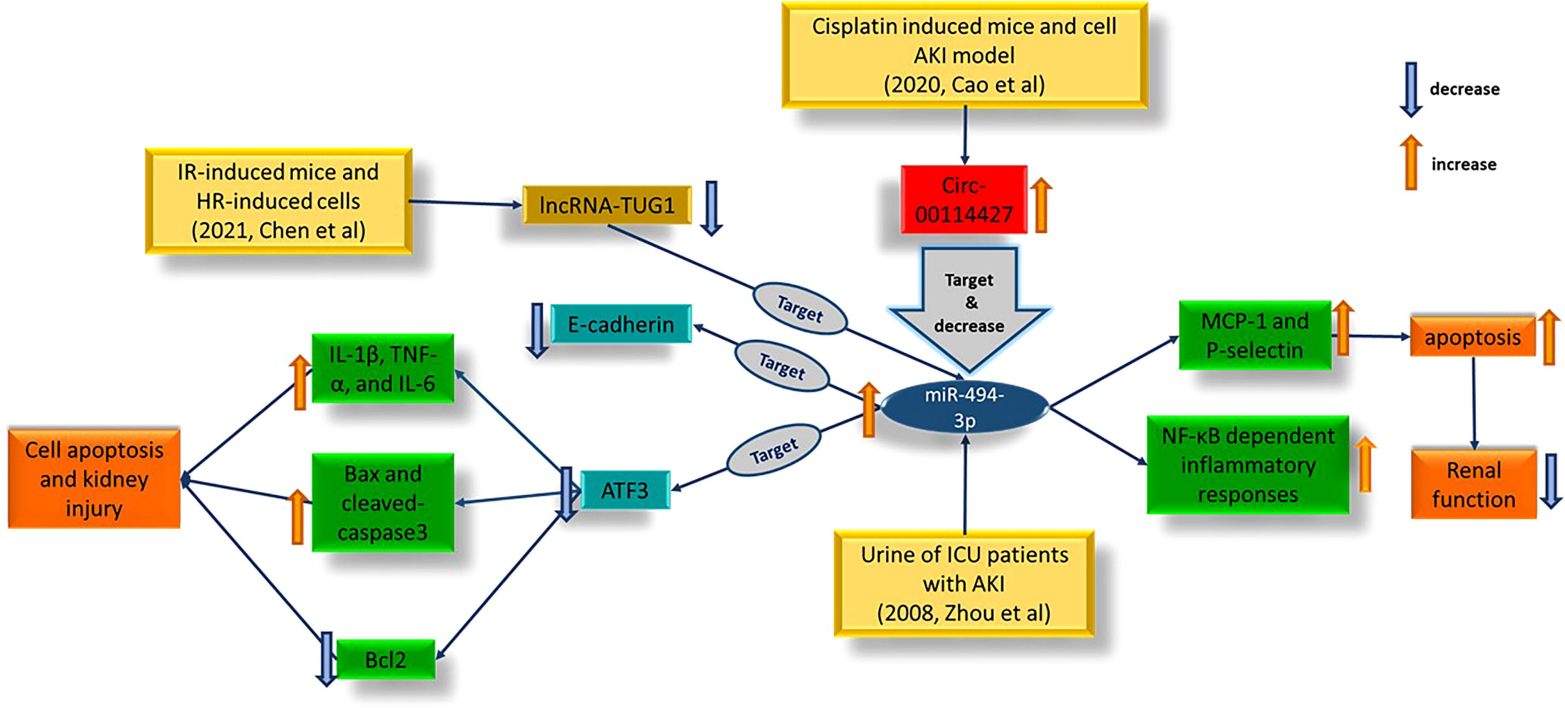
The probable pathway for controlling ischemic AKI.

In conclusion, combining the findings of various studies (as depicted in **Figure 9**) could elucidate specific pathways involved in regulating ischemic IRI. For instance, the role of up-regulation of miR-494-3p in the ICU AKI patients’ urine samples was previously detected (174). This research confirmed that an increase in miR-494-3p expression led to increased levels of MCP-1 and P-selectin, which in turn activated NF-κB related inflammatory immune responses, followed by apoptosis and decreased renal function. Alternative investigation conducted by Chen in 2021 (142), examined IR-induced mice and HR-induced HK-2, found that a decrease in lncRNA-TUG1 during AKI led to an increase in the amount of miR-494-3p. This, in turn, caused a decrease in E-cadherin and ATF3 levels, resulting in cell apoptosis and kidney injury (144). The study also showed that this process promoted the production of IL-1β, TNF-α, and IL-6 while increasing levels of Bax and cleaved-caspase3 and decreasing Bcl2. These findings suggest that the increase in ATF3 mRNA in extracellular vesicles present in urine may have a protecting signaling effect in target cells, through the uptake of extracellular vesicles and stimulation of ATF3-responsive gene expression programs (20).

The study by Cao et al., investigated the role of circ-0114427 in targeting miR-494-3p in both mice and cell cisplatin-induced AKI models. Their findings suggest that upregulating circ-0114427 can alleviate the injury caused by AKI by targeting the miR-494/ATF3/IL-6 pathway (162). These studies suggest that circ-0114427 might attend as a potential therapeutic agent for AKI, but further clinical research is necessary to validate this hypothesis.

## 6 Conclusion

The current human studies on ncRNA responses in AKI exhibit variability and lack reliable outcomes. This could be attributed to the diverse categories of pathogenesis observed in AKI or to the intricate miRNA-associated regulatory network that responds rapidly. As a result, further research is necessary to uncover the intracellular and molecular ncRNA regulatory mechanisms underlying ischemic AKI, in order to identify accurate predictors, biomarkers, and therapeutic agents. However, it is completely obvious that further clinical trials should be executed to elucidate the functional ability of ncRNAs in controlling ischemic AKI. Any attempt to use ncRNAs (especially miRNAs, and circRNAs) should consider the fact that any of these molecules might interfere in other pathophysiological processes and can be harmful.

By adding all the existing facts together and evaluating the mentioned studies, some of these molecules seem to act coordinately during ischemic AKI such as lncRNA-TUG1, miR-494-3p, and circ-00114427. These molecules might have more potential prognostic and therapeutic values due to their interference in different critical pathways.

## Author contributions

FS and AA are equally participating in writing the manuscript. All authors read and approved the final manuscript. All authors contributed to the article and approved the submitted version.
